# Differential and transferable modulatory effects of mesenchymal stromal cell-derived extracellular vesicles on T, B and NK cell functions

**DOI:** 10.1038/srep24120

**Published:** 2016-04-13

**Authors:** Mariano Di Trapani, Giulio Bassi, Martina Midolo, Alessandro Gatti, Paul Takam Kamga, Adriana Cassaro, Roberta Carusone, Annalisa Adamo, Mauro Krampera

**Affiliations:** 1 Stem Cell Research Laboratory, Section of Hematology, Department of Medicine, University of Verona, Italy

## Abstract

Mesenchymal stromal cells (MSCs) are multipotent cells, immunomodulatory stem cells that are currently used for regenerative medicine and treatment of a number of inflammatory diseases, thanks to their ability to significantly influence tissue microenvironments through the secretion of large variety of soluble factors. Recently, several groups have reported the presence of extracellular vesicles (EVs) within MSC secretoma, showing their beneficial effect in different animal models of disease. Here, we used a standardized methodological approach to dissect the immunomodulatory effects exerted by MSC-derived EVs on unfractionated peripheral blood mononuclear cells and purified T, B and NK cells. We describe here for the first time: i. direct correlation between the degree of EV-mediated immunosuppression and EV uptake by immune effector cells, a phenomenon further amplified following MSC priming with inflammatory cytokines; ii. induction in resting MSCs of immunosuppressive properties towards T cell proliferation through EVs obtained from primed MSCs, without any direct inhibitory effect towards T cell division. Our conclusion is that the use of reproducible and validated assays is not only useful to characterize the mechanisms of action of MSC-derived EVs, but is also capable of justifying EV potential use as alternative cell-free therapy for the treatment of human inflammatory diseases.

Mesenchymal stromal cells (MSCs) are multipotent stem cells that reside in many tissues, such as bone marrow (BM), adipose tissue, umbilical cord and amniotic fluid^1^,^2^,^3^,^4^. In addition to their proved capability to differentiate into mesodermal tissues *in vitro* and *in vivo*^5^, MSCs possess immunomodulatory properties elicited by inflammatory cytokines released in tissue microenvironment^6^,^7^. High levels of interferon-gamma (IFN-γ) and tumor necrosis factor-alpha (TNF-α) make MSCs to become immunosuppressive towards effector cells (IECs) of both innate immunity, including neutrophils, monocytes and natural killer (NK) cells and adaptive immunity, such as T and B cells^8^,^9^,^10^,^11^,^12^,^13^. MSC immunosuppression is linked to the release of several molecules, including transforming growth factor-β, indoleamine-2,3-dioxygenase (IDO), prostaglandin-E2, nitric oxide, and others^12^,^13^,^14^,^15^. These features were confirmed in different preclinical and clinical studies for a large spectrum of inflammatory and autoimmune disorders, such as Graft-versus-Host Disease (GvHD), cardiovascular diseases, liver diseases, autoimmune encephalomyelitis and sepsis^16^,^17^,^18^,^19^,^20^. During local inflammation, MSCs release various immunomodulatory and trophic molecules, generating a favorable microenvironment for tissue regeneration and the modulation of immune response, even in absence of cell engraftment^21^. Once injected into the animal, only a few MSCs reach the damaged tissues, while most of them remain entrapped in the lungs, spleen and liver^22^. Nevertheless, several groups have shown the beneficial effect of MSC-conditioned medium (CM) in different pathological conditions, i.e. by reducing cardiomyocyte apoptosis triggered by hypoxia/reoxygenation *in vitro*^23^, or preserving renal and kidney functions in rat models of diabetic renal injury and acute kidney injury (AKI)^24^,^25^.

It has been suggested that extracellular vesicles (EVs) can mediate the paracrine mechanism of MSCs, thus playing a role in tissue repair and immune regulation^26^.

EVs consist of different type of vesicles including: i. shedding vesicles or microvesicles (MVs) from plasma membranes (diameter ranging from 50 to 1000 nm) and expressing specific markers of the cell of origin; ii. exosomes, the smallest vesicles (diameter ranging from 40 to 100 nm) expressing tetraspanins (CD63, CD9), Alix and TSG101, originating inside cellular multivesicular endosome (MVE), and then secreted after fusion of these compartments with the plasma membrane; iii apoptotic bodies (diameter ranging from 50 to 5000 nm), which are secreted through blistering of apoptotic cell membranes surrounding histone proteins^27^. EVs are complex membranous structures composed of a lipid bilayer containing functional proteins, mRNAs and microRNAs (miRNAs)^28^. MiRNAs are a large family of small non-coding RNAs (22–24 nucleotides), which regulate gene expression by targeting specific mRNAs and, as a result, by inhibiting their translation towards proteins^29^. Recently, several groups have reported the involvement of EV-derived miRNAs in the context of immune modulation; for instance, MSCs may deliver miRNA-223 within EVs, which possess cardio-protective effects^30^. Similarly, an important role in the modulation of immune response has been assigned to miRNA-155 and miRNA-146^31^,^32^. Dendritic cells (DCs) modulate the response to endotoxin-induced inflammation by transferring miRNA-155 and miRNA-146 within exosomes. In particular, exosomal miRNA-155 promotes, while miRNA-146 reduce, inflammatory reaction in mice^33^.

Thus, EVs are considered as a physiological extracellular signaling system through which different cells interact reciprocally via a continuous release-uptake process.

The therapeutic effects of EVs have been investigated in different disease models, including cardiovascular diseases, acute kidney injury, and liver or lung injuries, where the injection of EVs resulted in an improvement of tissue damage and inflammation^34^,^35^,^36^,^37^,^38^,^39^. Some recent reports suggested the role of MSC-derived EVs in immunomodulation of different IECs; for instance, EVs co-cultured with unfractionated peripheral blood mononuclear cells (PBMCs) inhibited B cell proliferation and immunoglobulin release^40^,^41^, but non-univocal effects were observed on T cell proliferation^42^. However, qualitative and quantitative differences in EV-driven modulating effects could simply reflect the variability in the methodological approaches, i.e. isolation protocols, EV characterization and quantification, and immunological assays.

Here, we focused our attention on the EV-mediated interactions between resting or inflammatory primed MSCs and different IECs, either as unfractionated PBMCs or purified-T, -B and -NK cells, with the aim of quantifying the modulatory effect of EVs by using the standardized immunological assays normally employed to characterize MSC functions^43^. In addition, we assessed whether primed-EVs could activate resting MSCs and make them to become immunosuppressive towards T cells. Finally, we identified the presence of miRNA-155 and miRNA-146 within MSC-derived EVs, thus suggesting a potential role in their immunomodulatory activities.

## Materials and Methods

### Isolation and expansion of human MSCs and IECs

PBMCs were isolated from human blood using Lymphoprep (Stemcells Technologies). Purified IECs (CD3^pos^ T cells, CD19^pos^ B cells, and CD56^pos^ NK cells) were isolated from PBMCs using appropriate negative selection kits (Miltenyi Biotec) with at least 95% cell purity, as evaluated by flow cytometry.

MSCs from 14 different donors were isolated from BM aspirates of healthy donors (informed consent, approved by Ethical Committee of Azienda Ospedaliera Universitaria Integrata Verona; N. 1828, May 12, 2010 “Institution of cell and tissue collection for biomedical research in Onco-Hematology”). BM aspirates were cultured in 225 cm^2^ flasks at 1 × 10^5^ nucleated cells/cm^2^ concentration in alpha-minimal essential medium (α-MEM), 10% heat-inactivated fetal bovine serum (FBS), 100 U/mL penicillin, and 100 mg/mL streptomycin and 2 mM L-Glutamine (all from Sigma-Aldrich). After 72 hours, non-adherent cells were removed and the medium was replaced twice a week. Full characterization of MSCs has been already described by our group elsewhere^43^^,^^47^.

MSCs were detached (0.05% Tripsin-EDTA; Gibco) and harvested when 80% confluent, and then either reseeded at 1 × 10^3^/cm^2^ concentration or frozen until use. All experiments were performed between passages 2 and 7.

In all experiments, MSCs at 80% confluence were treated or not for 40–48 hours with 10 ng/mL IFN-γ and 15 ng/mL TNF-α (R&D Systems) to induce the inflammatory priming, as previously described by or group elsewhere^46^^,^^47^.

### Purification of MSC-derived EVs

CM from MSC culture at 80% confluence was aspirated, cells were washed with phosphate-buffered saline (PBS) to remove the residual fetal bovine serum (FBS), and fresh culture medium supplemented with 10% EV-depleted FBS, obtained through 18 hour-centrifugation at 100.000 g was added. After 2 days of incubation, CM from MSCs previously treated or not with inflammatory cytokines was collected and underwent different steps of centrifugation, as previously described by other groups^44^,^45^. Briefly, CM was centrifuged for 10 minutes at 300 g, 30 minutes at 4 °C at 2000 g to remove cell debris and apoptotic bodies, and then 90 minutes at 4 °C at 100.000 g to collect EVs. The pellet was washed with PBS and underwent another step of ultracentrifugation at 100.000 g for 90 minutes at 4 °C to concentrate and purify EVs, which were then resuspended in PBS for immunological assays or stored at −80 °C.

### EV characterization and quantification

Instrument calibration to detect EVs was performed by comparing them with different fluorescent latex beads by flow cytometry on BD FACSCanto II. Beads of different size, 0.1 μm, 0.2 μm, 0.5 μm and 1.0 μm (Life Technologies) were mixed with EVs to generate an analytic gate for the following experiments.

EV quantification was obtained with two different methods. EVs were quantified by Trucount Tubes (BD Biosciences) to obtain the absolute numbers. Each tube contained a lyophilized pellet that once resuspended released a known number of 4.3 μm beads. The tubes were used according to manufacturer’s recommendations and the absolute count was calculated by using the following formula: (number of events in the EV-containing gate/number of events in the bead-containing gate) × (number of beads per test/volume). To eliminate noise events, 0.22 μm-filtered PBS was analyzed under identical conditions and the number of events was subtracted from each analysis. EV protein content was determined by Quantum Micro Protein method (EuroClone).

To confirm particle size, purified EVs were analyzed by Nanoparticle Tracking Analysis (NTA) using NanoSight NS300 model (Malvern).

For immunophenotypic analysis, EVs were adsorbed to 3.9 μm latex beads (Life Technologies). Briefly, 5 μg of resting or primed EVs were mixed with 10 μl of latex beads for 15 minutes at room temperature. Then, 1 ml of PBS was added to each sample and incubated in a rotating wheel overnight. Next, 110 μl of glycine 1 M was added to the sample and mixed on the bench at room temperature for 30 minutes. Bead-bound EVs were centrifuged for 3 minutes at 4000 rpm, pellets were washed in PBS/0.5% BSA (bovine serum albumin) for three times and resuspended in 0.5 ml of PBS/0.5% BSA. Finally, 10 μl of bead-bound EVs were stained with specific antibodies for 30 minutes at 4 °C. For the staining, the following monoclonal antibodies against human markers were used: IgG1k-PE, CD73-PE, CD90-PE, CD105-PE, CD54-PE (ICAM-1), CD106-PE (VCAM-1), HLA-ABC-PE, HLA-DR-PE and CD63-PE all from BD Biosciences, IgG2b-PE and CD274-PE (programmed death-ligand 1 or PD-L1) from Biolegend. All tubes were washed and resuspended in 200 μl of PBS/BSA 0.5%. Data analysis was conducted using FlowJo software (TreeStar).

### Immunological assays

To assess MSC immunomodulatory capabilities on different IECs, standardized assays were carried out as previously described by our group^43^,^46^. Resting or primed-MSCs were cultured in each well with IECs at either 2 × 10^4^ cell concentration (high ratio, corresponding to a confluent monolayer), or 4 × 10^3^ or 2 × 10^3^ cell concentration (low ratio) in 96 well plates. After MSC adhesion, 2 × 10^5^ T cells, 2 × 10^4^ B cells, or 2 × 10^4^ NK cells, previously stained with 5 μM carboxyfluorescein succinimidyl ester (CFSE) or Violet Cell Trace from Life Technologies, were added.

PBMCs were stimulated with 5 μg/ml of phytohemagglutinin (PHA) for 4 days in Iscove Modified Dulbecco Medium (IMDM) supplemented with 10% pooled human AB serum. T cells were activated with 0.5 μg/mL cross-linking anti-CD3 and anti-CD28 antibodies (Sanquin) for 6 days in Roswell Park Memorial Istitute (RPMI) supplemented with 10% human AB serum. B cells were activated with 5 μg/mL F(ab’)^2^ anti-human IgM/IgA/IgG (Jackson Immunoresearch), 50 IU/mL rhIL-2 (Proleukin; Novartis), 50 ng/mL polyhistidine-tagged CD40 ligand, 5 μg/mL anti-polyhistidine antibody (R&D Systems), and 0.5 μg/mL CpG ODNs (Invivogen) for 4 days in RPMI supplemented with 10% FBS (Invitrogen Life Technologies). NK cells were activated by 100 IU/mL rhIL-2 for 6 days in IMDM supplemented with 10% human AB serum.

To test whether paracrine factors were involved in immunomodulatory mechanism, Transwell^®^ 24 system with a 0.4 μm pore size (BD Biosciences) was used keeping the same MSC:IEC ratio.

In all the experiments, cells were harvested at the end of co-culture and stained with PerCP-Vio700 or Vioblue mouse anti-human CD45 monoclonal antibody, CD4-APC-Vio770 and CD8-PE-Vio770 (Miltenyi Biotec), and TOPRO-3 Iodide (Life Technologies); the proliferation was assessed on viable TOPRO-3^neg^ CD45^pos^ cells by FlowJo software (TreeStar) as the percentage of cells undergoing at least one cell division. The proliferation rate was obtained according to the following formula: (percentage of CD45^pos^ cell proliferation with MSCs)/(percentage of CD45^pos^ cell proliferation without MSCs) × 100.

### Evaluation of EV-mediated immunomodulation

To evaluate the EV role in immune regulation, the following specific inhibitors of EV biogenesis were added to MSC cultures: 10 μM GW4869 (Sigma Aldrich), 10 μM imipramine (Sigma Aldrich), or 60 μM DEVD (Sigma Aldrich). Briefly, complete medium of MSCs at 80% confluence was removed and replaced with medium containing inhibitors. After 2 days, the medium was replaced with fresh culture medium supplemented with 10% EV-depleted FBS and EV number was evaluated to validate the effect of inhibitors. For the immunomodulatory assays, MSCs were washed twice in PBS to remove residual inhibitors, and the immunomodulation was detected using the same co-culture assays described above.

For the same purpose, 1 × 10^4^ stimulated-IECs were cultured alone or in presence of 3 × 10^6^ of resting and primed-EVs, whose proliferation rate was analyzed after 4 days (for PBMCs, purified T and B cells) or 6 days (for NK cells).

To dissect the mechanism of EV-mediated priming, 2 × 10^4^ MSCs were cultured with 2 × 10^6^ resting or primed-EVs in 96 well plates. Untreated or IFN-γ/TNF-α-treated MSCs were used as controls. After 48 hours, CM was removed and each well was washed twice with PBS to remove residual cytokines. The EV-mediated priming was evaluated by immunophenotypic analysis and the standardized immunomodulation assays previously described.

### EV-uptake assay and immunofluorescence

To assess EV internalization by IECs, MSC membranes were stained with 2×10^−6^ M of PKH26 PKH26 Red Fluorescent dye (Sigma-Aldrich) according to manufacturer’s recommendations. Then, PKH26-labeled or -unlabeled MSCs were cultured in presence of IECs and EV uptake was assessed after 1, 2 or 4 days. At the end of co-culture, cells were detached by trypsin and stained with the following monoclonal antibodies: CD45-Vioblue (Miltenyi Biotec), CD3-V500 (BD Biosciences), CD4-APC-Vio770, CD8-FITC, CD14-FITC (Miltenyi Biotec), CD16-PercP-Cy5, CD19-PE-Cy7 (BD Biosciences) to identify the different IEC population, while TOPRO-3 was used to identify viable cells. The internalization of MSC-derived EVs by IECs was analyzed by FACS analysis.

To further confirm the transfer of EVs into IECs, cells were analyzed at the end of co-culture by confocal microscopy. Briefly, cells were detached by trypsin and stained with Viobright-FITC anti-human CD45 monoclonal antibody (Miltenyi Biotec). Then, cells were fixed using Cytofix/Cytoperm kit (BD Biosciences) and TOPRO-3 (Invitrogen Life Technologies) was used to reveal nuclei. Finally, cells were loaded into the CytoSpin centrifuge’s sample chamber and centrifuged 5 minutes at 400 rpm.

Images were obtained by LSM 710 confocal microscopy (Zeiss) at 63x magnification and elaborated with ZEN imaging software (Zeiss).

### Statistical analysis

Data were expressed as mean ± SEM. Statistical analysis was performed by Prism software (GraphPad) using the Wilcoxon test to compare differences between EVs from resting and primed-MSCs, one-way ANOVA test was used to assess the differences in immumodulatory effects, while two-sample *t* test were used to evaluate the differences of miRNA expression. P < 0.05 was considered statistically significant.

## Results

### MSC-mediated immunomodulation is driven by paracrine factors

**Figure 1:**
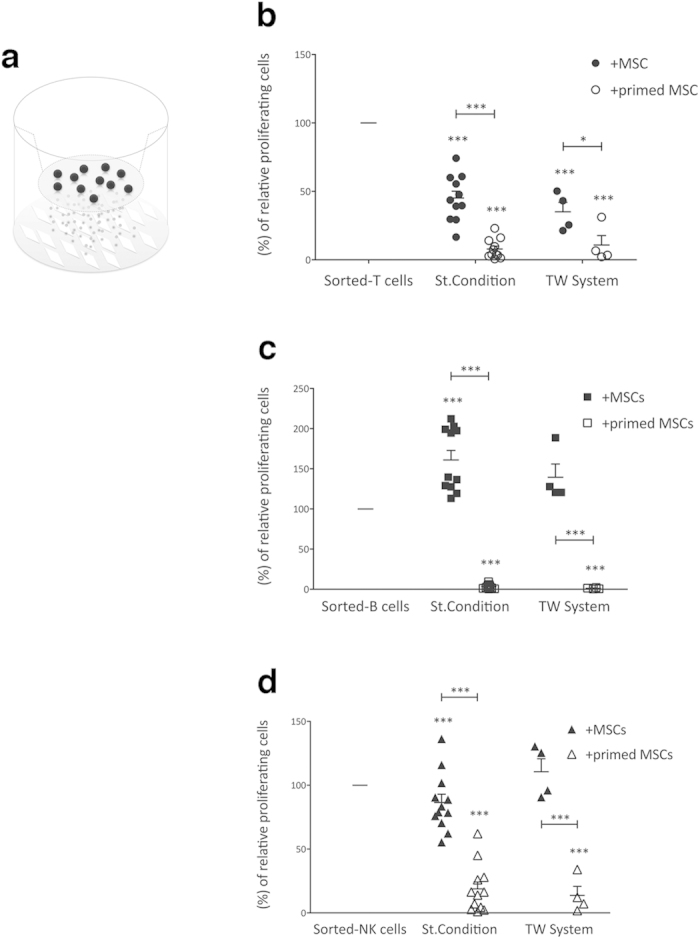
MSC immunomodulation is mediated by paracrine molecules. (**a**) Schematic representation of Transwell^®^ system with MSCs in the bottom well and IECs in the top well. A 0.4 μm-porous membrane was used to prevent cell-cell interaction and permit soluble molecule exchange. Sorted-IECs (T, B and NK cells) were stimulated with specific stimuli and cultured alone or in the presence of resting or primed allogeneic MSCs. At the end of co-culture, IEC proliferation was assessed using carboxyfluorescein succinimidyl ester (CFSE) dilution method, as described in Materials and Methods section. CFSE fluorescence was analyzed after 6 days for T (at 10:1 T/MSC ratio) and NK (at 1:1 NK/MSC ratio) cells (**b**,**d**, respectively), while for B cells (**c**) the fluorescence was detected after 4 days of co-culture (at 1:1 B/MSC ratio). The same IEC:MSC ratios were maintained to assess the effect of MSC paracrine molecules on sorted-T, -B and -NK cells (**b**–**d**, respectively) proliferation by use of Transwell^®^ 24 system. The results are expressed as relative proliferation percentage of IECs, normalized to IEC cultured alone (100%). Error bars represented mean ± SEM of twelve independent experiments for standard immunological assays and four independent experiments for Transwell^®^ assays. ^***^P < 0.001.

We assessed first whether the immunomodulatory properties of MSCs in close contact with IECs were comparable to the effects exerted by their paracrine signals. To this aim, resting or primed MSCs were cultured in presence of IECs both in standard conditions and in Transwell^®^ system, thus preventing cell-to-cell contact but not the exchange of soluble molecules (Fig. 1a and Supplemental Fig. S1).

In both co-culture systems, resting MSCs exerted a stronger suppressive effect on T cells as compared to the other lymphocyte populations (Fig. 1b). These differences were related to the level of inflammatory cytokines released by IECs, as previously discussed^7^. Accordingly, B cell division was not inhibited by resting-MSCs in both co-culture settings, due to their inability to induce MSC licensing^12^ (Fig. 1c). In the absence of inflammatory stimuli, NK cell co-culture led to a moderate activation of MSCs, which in turn determined a mild (15%) inhibition of NK cell proliferation in standard culture conditions, even lower in Transwell^®^ system (Fig. 1d). However, following pre-treatment with IFN-γ and TNF-α, MSCs acquired a significant immunosuppressive effect, reducing T, B and NK cell proliferation by more than 80% in both co-culture approaches (Fig. 1b–d).

These results are in agreement with the well-known concept that the immunosuppressive features of human MSCs are mostly cell-to-cell contact-independent^7^, thus suggesting a possible role for EVs in intercellular signaling through active molecule delivery.

### Different uptake of MSC-derived EVs by IECs

**Figure 2:**
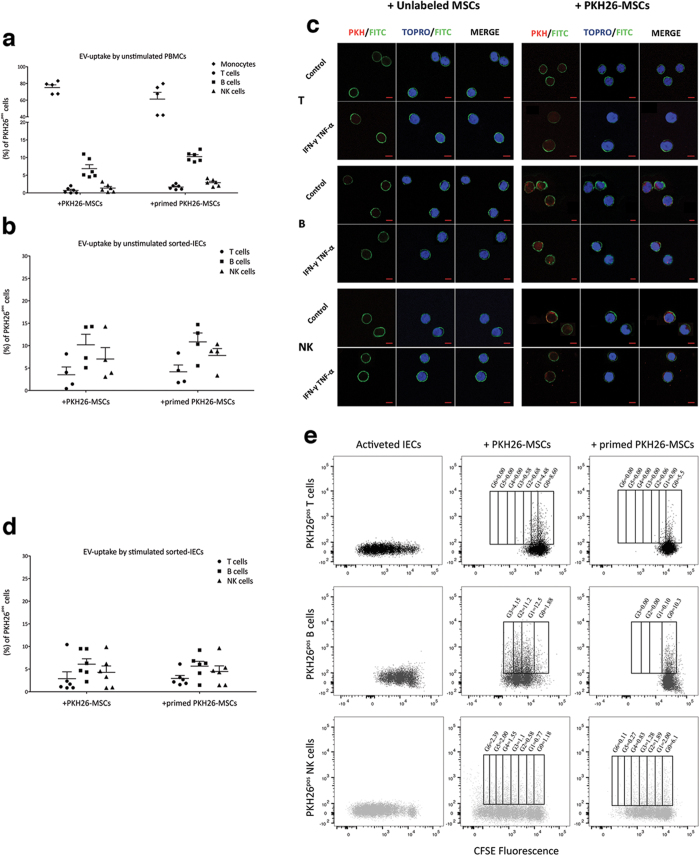
Internalization of MSC-derived EVs by IECs. Resting and primed PKH26-MSCs were cultured in presence of unstimulated PBMCs or sorted-T, -B or -NK cells in order to assess the transfer of MSC-derived EVs to IECs. After 4 days, the cells were harvested and labeled with anti-CD45, anti-CD3, anti-CD14, anti-CD56, anti-CD19 to identify the different IEC lineage inside unfractionated PBMCs (**a**); anti-CD45 were used for sorted-IECs (**b**–**e**). TOPRO-3 was added to detect viable cells. The EV-uptake by IECs was detected as percentage of lineage specific^pos^/PKH26^pos^ IECs by FACS. (**c**) Representative immunofluorescence staining of CD45^pos^/PKH26^pos^ IECs. At the end of co-cultures, cells were detached and labeled with anti-CD45 (green) and TOPRO-3 (blue nuclei) to assess the incorporation of PKH26-EVs (red). Scale bars: 5 μm. Images were obtained by LSM 710 confocal microscopy (Zeiss) at 63x magnification. (**d**) EV-internalization by stimulated CFSE labeled IECs was evaluated after 6 days for T and NK cells and after 4 days for B cells. (**e**) CFSE plot representative of three independent experiments, showing the localization of EVs inside IEC generation as percentage of CFSE^pos^/PKH26^pos^ IECs. Error bars represented mean ± SEM.

To assess whether the communication between MSCs and IECs could be driven by the exchange of EVs, MSCs labeled or not with PKH26 were co-cultured with unlabeled IECs (Fig. 2 and Supplemental Fig. S2).

When PKH26^pos^ MSCs, either resting or inflammatory-primed, were co-cultured with unfractionated PBMCs, EVs were mostly internalized by monocytes and scarcely by lymphocytes after 24 hours (Supplemental Fig. 2a) up to day 4 (Fig. 2a); in fact, at the end of co-culture the percentage of PKH26^pos^ monocytes was 75.11 ± 3.24 in presence of resting PKH26^pos^ MSCs and 61.27 ± 8.11 in the presence of inflammatory-primed PKH26^pos^ MSCs (Fig. 2a). Among lymphocyte subsets, CD19^pos^ B cells displayed the highest EV uptake (6.86 ± 10.26%) as compared to CD56^pos^ NK cells (1.35 ± 0.46%) and CD3^pos^ T cells (0.702 ± 0.30%) in presence of resting MSCs. EV internalization by unselected lymphocytes increased following inflammatory priming (10.26 ± 0.56% for B cells, 2.90 ± 0.38% for NK cells and 1.65 ± 0.27% for T cells). Within T cell subsets, EV internalization was similar in CD4^pos^ and CD8^pos^ T cells, and slightly induced by inflammatory priming (Supplemental Fig. S2b).

By contrast, EV uptake was significantly more evident when using purified IECs, especially T and NK cells (Fig. 2b and Supplemental Fig. S2c). For instance, by comparing the same subset of lymphocytes in two different experimental settings, we observed a 5.00-fold increase of PKH26^pos^ purified T cells in presence of resting PKH26^pos^ MSCs and a 2.60-fold increase in presence of primed PKH26^pos^ MSCs, as compared to PKH26^pos^/CD3^pos^ T cells from unfractionated PBMCs. Similarly, purified NK cells showed a 5.20-fold and 2.70-fold increase in PKH26-fluorescence in presence of resting and primed PKH26^pos^ MSCs, respectively. Purified B cells maintained the highest degree of EV uptake as compared to purified T and NK cells, but showed only a slight increase of EV uptake by both resting (1.50-fold) and primed (0.10-fold) PKH26^pos^ MSCs as compared to PKH26^pos^/CD19^pos^ B cells from PBMCs in the same co-culture conditions. Confocal microscopy confirmed the internalization of MSC-derived EVs, thus excluding their non-specific adhesion to cell membrane (Fig. 2c).

We then assessed whether pre-activated IECs maintained the same capability of internalizing EVs (Fig. 2d). Again, the levels in EV-uptake were maintained in all purified IEC types, showing the highest capability for B cells as compared to NK and T cells. Intriguingly, when resting and primed PKH26^pos^ MSCs were co-cultured with activated CFSE-labeled IECs, we observed a significant distribution of PKH26^pos^ EVs inside the early cell progeny, thus suggesting that EVs could affect IEC proliferation (Fig. 2e).

When resting PKH26^pos^ MSCs were co-cultured with T cells, PKH26^pos^ T cells were mainly inside the populations in the G0 and G1 phases of cell cycle, but following inflammatory priming the EVs were present only in G0 cell population.

When resting PKH26^pos^ MSCs were co-cultured with B cells (control condition), the latter were not inhibited and PKH26^pos^ B cells were equally distributed in each cell generation. Conversely, B cells co-cultured with primed MSCs were significantly inhibited and became strongly positive for PKH26^pos^ EVs. Similarly, NK cells were only slightly suppressed when cultured with resting MSCs and displayed homogenous distribution of PKH26^pos^ EVs in all cell generations. However, NK cells were significantly affected by primed MSCs and their inhibition paralleled with the localization of EV uptake mainly in G0 and G1 phases of cell cycle.

Overall, our data show that the uptake of MSC-derived EVs occurs in both resting and, mostly, activated IECs, thus highlighting a possible role for EVs in immunosuppression.

### Immunomodulatory properties of MSC-derived EVs

To investigate the immunomodulatory effects of MSC-derived EVs on IEC proliferation, specific inhibitors of EV secretion were added to MSC cultures at doses that did not alter MSC viability and morphology (data not shown). Thus, MSCs were treated for 48 hours before supernatant collection with the following compounds: i. GW4869, an inhibitor of sphingomyelinase 2, which is involved in the production of the intraluminal vesicles into MVE, thus impairing exosome release; ii. imipramine, an inhibitor of microvesicle shedding; iii. DEVD, an apoptosis inhibitor that prevents the production of apoptotic bodies.

**Figure 3:**
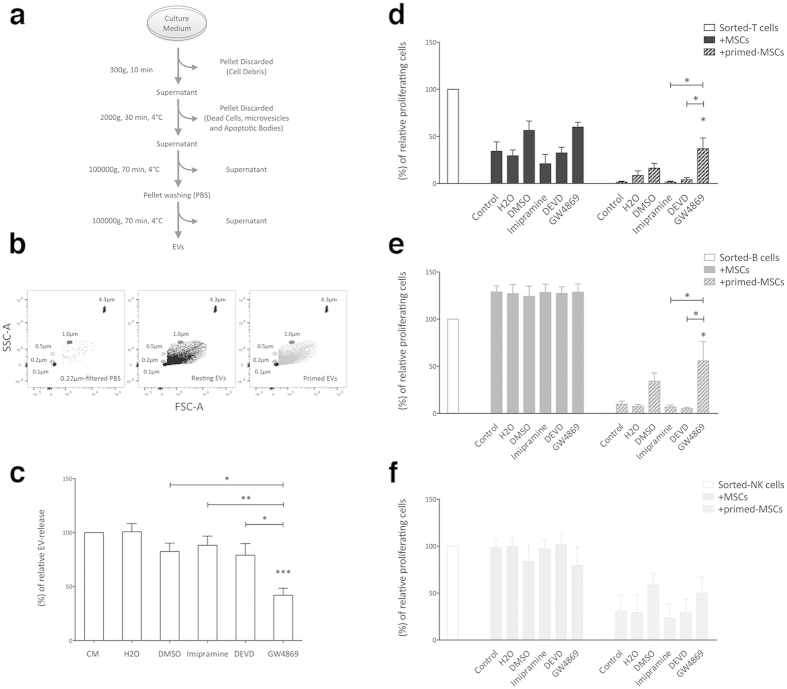
Inhibition of EV secretion impairs MSC immunosuppression. (**a**) Schematic representation of EV isolation protocol. (**b**) EV gate was carried out by using size-calibrated fluorescent beads ranging from 0.1 μm to 0.9 μm. The number EVs were calculated using 4.3 μm TruCount beads, which are shown in the upper right corner. The absolute count of EVs was subtracted to background noise events from 0.22 μm-filtered PBS. (**c**) Count of EVs obtained from MSCs treated for 48 hours with 10 μM GW4869, 10 μM imipramine, 60 μM DEVD and relative control vehicles, including: DMSO for GW4869 and DEVD; H2O for Imipramine. The results are expressed as percentage of relative EV-release inhibition, normalized to number of EVs obtained from untreated MSCs (100%). Resting and primed MSCs treated with GW4869, imipramine and DEVD were cultured in presence of activated CFSE labeled T, B and NK cells (**d**–**f**, respectively) in order to assess the effects of the inhibition of EV release on the immunomodulatory properties of MSCs. The results are expressed as relative proliferation percentage of IECs, normalized to IEC cultured alone (100%). Error bars represented mean ± SEM of four independent experiments. ^*^P < 0.05, ^**^P < 0.01, ^***^P < 0.001.

First, EVs were isolated by using differential centrifugation (Fig. 3a) and quantified by FACS analysis to confirm the effects of each inhibitor. Beads of different size (0.1 μm, 0.2 μm, 0.5 μm and 1.0 μm) were used for instrument calibration, defining the upper and the lower limits of EV gate with 1.0 μm and 0.1 μm beads, respectively (Fig. 3b). EV concentration was calculated by using Trucount Tubes containing a known number of 4.3 μm beads. GW4869 significantly reduced the EV release by more than 50%. By contrast, imipramine and DEVD (Fig. 3c) showed a lower inhibition, probably due to the EV isolation strategy (Fig. 3a).

MSCs were then treated with the inhibitors and cultured in presence of T, B and NK cells to evaluate the EV involvement in immunomodulation (Fig. 3d–f, respectively). As expected, only GW4869 significantly restored T and B cell proliferation in presence of primed-MSC. Conversely, exosome inhibition reverted only partially the effect of resting-MSCs on T cells and was completely ineffective on NK cell proliferation.

Finally, we never detected any effects on IEC proliferation either in presence of resting or primed MSCs by inhibiting vesicle shedding and apoptotic body release.

On the whole, our observations suggest that the immunosuppressive effects of MSCs can be mediated, at least in part, by EV secretion.

### Characterization of MSC-derived EVs

**Figure 4:**
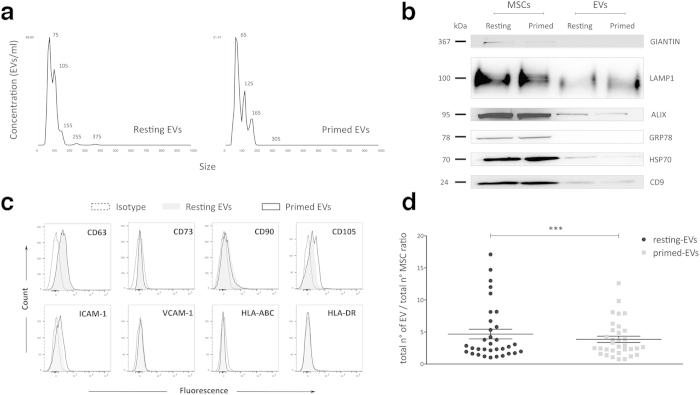
Characterization of MSC-derived EVs. (**a**) Size distribution of resting and primed EVs obtained by NTA. (**b**) Immunoblot analysis of Giantin, LAMP1, Alix, GRP78, HSP70 and CD9 expression in resting and primed MSCs and purified EVs. This blot is representative of three independent experiments showing the same trends. (**c**) Representative plots of the immunophenotypic analysis of MSC-derived EVs showing the expression profile of a specific exosome marker (CD63), mesenchymal stromal cell markers (CD73, CD90 and CD105), adhesion molecules (ICAM-1 and VCAM-1) and MHC class I and II (HLA-ABC and HLA-DR, respectively). The histograms display the isotopic controls (dotted curve) and specific markers of resting (filled curve) and primed (black curve) EVs. (**d**) Graph showing quantitative differences of EV release between resting and primed MSCs. Data represented as mean ± SEM of ratio between number of EVs and number of cells of origin obtained from 33 independent experiments. ^***^P < 0.001.

Next, we performed a qualitative and quantitative analysis of MSC-derived EVs. NTA showed that the size of isolated EVs ranged between 60 nm and 150 nm, corresponding to exosomes, while only a few peaks ranged from 200 nm and 400 nm, corresponding to larger EVs (Fig. 4a).

Biochemical analysis revealed the presence of specific exosome markers, such as CD9, Alix, LAMP1 and HSP70, both in resting and primed EVs. Moreover, to exclude the presence of potential contaminants, we verified the absence of Giantin and GRP78, which specific molecules of Golgi apparatus and endoplasmic reticulum, respectively (Fig. 4b). Furthermore, immunophenotyping revealed the expression of CD63, an exosome marker, both in resting and primed-EVs (Fig. 4c). As for mesenchymal molecules, EVs expressed CD90 and CD105, but not CD73; the expression of CD54 (ICAM-1), which is an adhesion molecule involved in the tethering of EVs to target cells^28^, was weak in EVs from resting MSCs, but increased following inflammatory priming; by contrast, CD106 (VCAM-1) was undetectable in resting EVs and weakly expressed in primed EVs at the levels observed in MSCs^47^. Differently from MSCs, resting and primed EVs never expressed MHC class I (HLA-ABC) and II (HLA-DR).

As the quantity of released EVs depends on the initial MSC number, EVs were quantified as EV:MSC ratio to compare EVs obtained from resting and primed MSCs (Fig. 4d). The number of EVs secreted by resting MSCs was significantly higher than that from primed MSCs (n = 35), without significant differences among different cell passages and donors (Supplemental Fig. S3).

### Immunosuppressive effect of EVs on IEC proliferation

**Figure 5:**
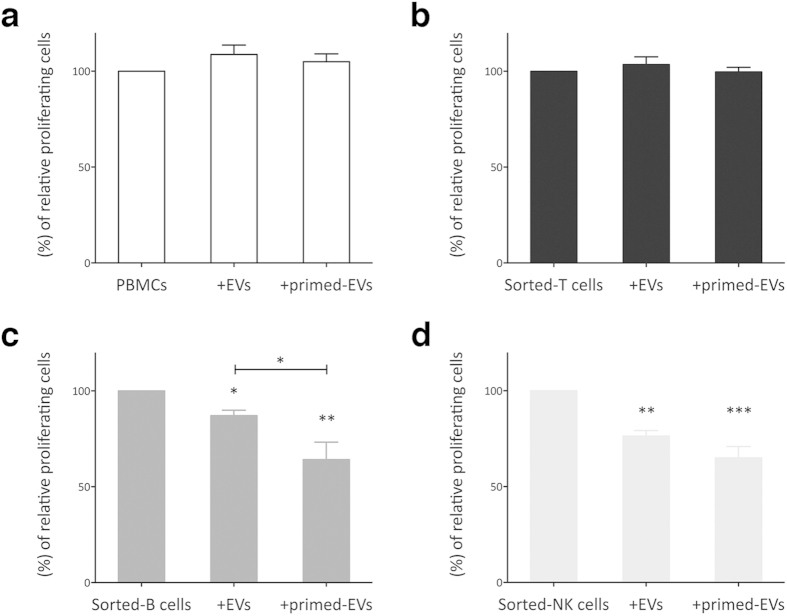
Immunosuppressive properties of MSC-derived EVs. EVs were purified from resting and primed MSCs and added to unfractionated PBMCs or sorted-T, B and NK cells that were activated by specific stimuli (1 × 10^4^:3 × 10^6^ IEC:EV ratio). At the end of co-cultures, the cells were harvested and IEC proliferation was assessed by FACS analysis. CFSE fluorescence was analyzed after 4 days for PBMCs, T and B cells (**a**–**c**, respectively), while for NK cells (**d**) the fluorescence was analyzed after 6 days of co-culture. The results are expressed as relative proliferation percentage of IECs, normalized to IEC cultured alone (100%). Error bars represented mean ± SEM of six for PBMCs, and five for sorted-T, NK cells and B cells independent experiments. ^*^P < 0.05, ^**^P < 0.01, ^***^P < 0.001.

PKH26^pos^ EVs were cultured in presence of PBMCs to verify the ability of IECs to internalize purified EVs (Supplemental Fig. S4a). We observed a trend of EV internalization by the different IEC subpopulations, similarly to that previously shown for MSC:IEC co-culture (Fig. 2). Thereafter, to validate the immunomodulatory effects of MSC-derived EVs, purified resting and primed EVs were added to IEC cultures. First, IECs were exposed to increasing quantities of EVs (data not shown) to identify the most suitable EV:IEC ratio for the standardization of the immunological assays. In the following experiments all IECs were treated with the same number of EVs (3 × 10^6^ particles/1 × 10^4^ IECs) obtained from resting or primed MSCs (Fig. 5). When assessing EV-dependent immunomodulation on PHA-stimulated PBMCs, no inhibitory effect on CD45^pos^ cell proliferation was observed (Fig. 5a); similar results were obtained by analyzing only CD3^pos^ cells by gating strategy on PBMCs (Supplemental Fig. S4b).

Then, we tested the immunosuppressive effect of EVs on purified T, B and NK cells on the basis of the evidence that sorted IECs showed a higher EV uptake as compared to unfractionated PBMCs (Fig. 2). Similarly to what observed with CD3^pos^ PBMCs, T cells were not inhibited in presence EVs obtained from resting or primed MSCs (Fig. 5b), but a slight increase in CD4^pos^ cells was observed as compared to sorted T cells cultured alone (Supplemental Fig. S4c). Conversely, resting EVs displayed a significant suppressive effect on B and NK cell proliferation that became more evident following inflammatory priming in both lymphocyte populations (Fig. 5c,d). Although cytokine priming induced in primed MSCs the overexpression of IDO, the main immunosuppressive enzyme involved in MSC immunosuppression^48^, EV-mediated immunosuppression was IDO-independent, as demonstrated by the totally absence of this molecule within resting and primed EVs (Supplemental Fig. S4d). Conversely, resting and primed EVs expressed PD-L1 (CD274) on their surface, which is a well-known immunosuppressive molecule (Supplemental Fig. S4e)^49^,^50^. Finally, we evaluated the presence of miRNAs within MSC-derived EVs. The profiling of small RNAs isolated from EVs and MSCs by miRNA microarray analysis showed that EVs had higher level of miRNAs as compared to MSCs (Supplemental Fig. S5a). Moreover, we observed that inflammatory priming induced the increase in the level of miRNA-155 and miRNA-146 (Supplemental Fig. S5b), which are two miRNAs involved in the activation and inhibition of inflammatory reactions^31^,^32^,^33^.

### Primed EVs make resting MSCs to become immunosuppressive towards T cells

**Figure 6:**
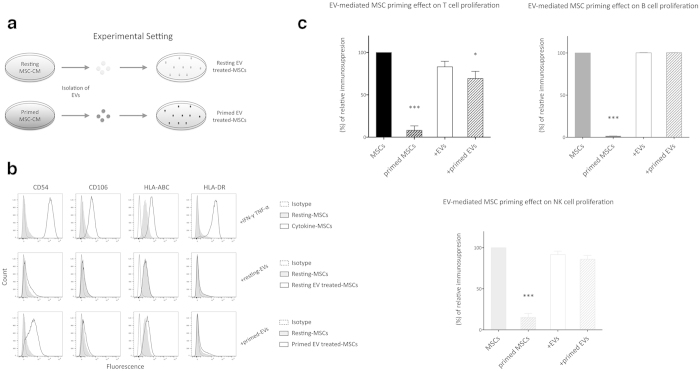
Effect of EVs on immunomodulatory properties of MSCs. (**a**) Methodological approach to induce MSC priming with EVs (as described in Materials and Methods section). (**b**) Immunophenotypic analysis of MSCs exposed to resting or primed EVs. IFN-γ/TNF-α-treated MSCs were used as positive control. The histograms show the isotopic controls (dotted curve) and specific markers of control (filled curve) or treated (black curve) MSCs derived from three independent experiments. (**c**) Resting and primed EVs were incubated with resting MSCs to induce the immunosuppressive phenotypic switch. After 48 hours, sorted-T, -B and –NK cells were added to MSC cultures to assess their immunomodulatory properties. IFN-γ/TNF-α-treated MSCs were used as positive control. CFSE fluorescence was analyzed after 6 days for T and NK, while for B cells the fluorescence was analyzed after 4 days of co-culture. The results are expressed as percentage of relative increase in MSC immunosuppression, normalized to the effect of untreated MSC on IECs (100%). Error bars represented mean ± SEM of five independent experiments. ^*^P < 0.05, ^***^P < 0.001.

Taken together, our data indicate so far that MSCs secrete immunologically active EVs that are capable of modulating B and NK cell proliferation similarly to what observed with entire MSCs. However, EV blocking assays using EV inhibitors are consistent with an effect of MSC-derived EVs also on T cell proliferation (Fig. 3d). Thus, we hypothesized an indirect mechanism of immunosuppression triggered by EV-mediated priming of resting MSCs. To this aim, we incubated resting MSCs with 2 × 10^6^ EVs derived from either resting or primed MSCs (Fig. 6a). After 48 hours of incubation primed EVs induced an immunophenotypic switch similar to that observed in IFN-γ- and TNF-α-treated MSCs (Fig. 6b), i.e. higher expression of CD54 (4.80-fold), HLA-ABC (1.51-fold) and HLA-DR (2.10-fold) as compared to resting MSCs. None of these immunophenotypic changes were induced in MSCs treated with resting EVs, except for the slight increase in CD54 expression (2.90-fold). Furthermore, the treatment with resting and primed EVs induced the over-expression of IDO mRNA (Supplemental Fig. S5c).

To assess whether the EV-mediated priming could enhance immunosuppression too, MSCs treated with resting or primed EVs were cultured in presence of purified T, B and NK cells and compared with resting and IFN-γ- and TNF-α-primed-MSCs. Interestingly, MSC exposure to primed EVs induced a significant increase in the immunosuppressive effect on T cells, but not on NK and B cells (Fig. 6). Conversely, the treatment with resting EVs did not change the degree of MSC immunomodulation.

## Discussion

EVs are shuttles of bioactive molecules, playing a role as information transporters through their internalization by target cells, thus eliciting different responses. EV effects are not limited to adjacent cells, but EVs may exert their activity in distant tissues. Most cell types produce EVs capable of transmitting multiple immunological signals. EVs may trigger the immune system depending on their molecular composition and cells of origin, not only by carrying peptide-loaded MHC molecules or tumor and pathogen antigens to antigen presenting cells^51^,^52^, but also by dampening inflammatory response through the exposition of pro-apoptotic molecules, such as CD95L, TRAIL or Galectin-9^53^,^54^.

Many studies have confirmed the beneficial role of EVs *in vivo*, thus paving the way for alternative, replicating-cell-free, therapeutic approaches in inflammatory and autoimmune diseases, by circumventing some drawbacks related to cellular therapy, such as tumor development^26^. In addition, many reports have shown variable effects of EVs on activated T and B cells^40^,^41^,^42^, but the results of those studies are not easily comparable due to the different methodological approaches, related to both the protocols of purification and quantification of EVs and the experimental read-out to assess their immunomodulatory effects on IECs. Immunological assays are essential to identify and measure the influence of third-party cells or cell products; however, there are several critical variables that can lead to different results, such as the use of purified IECs rather than unfractionated PBMCs, cell ratios in co-culture systems, type and duration of cell stimulation, cell viability and proliferation rate assessment^55^. Although the use of unfractionated PBMCs, containing a combination of lympho-monocytic cells, represents a powerful tool to study the overall immunomodulatory effect, the presence of different cell subsets at variable concentrations, depending on cell donors, frequently generates variable and poorly reproducible data.

In this study, by using purified IEC-based immunological assays previously validated in cooperative studies^43^,^56^, we showed that the ability of MSCs to regulate IEC proliferation is almost fully driven by paracrine mechanisms, as MSCs and IECs can communicate through the exchange of EVs that possess comparable immunosuppressive effect to their cells of origin. The degree of EV-mediated immunomodulation seemed to be proportional to the ability of each IEC population to uptake EVs. Accordingly, B cells were mostly prone to incorporate EVs and to be modulated, as compared to other lymphocyte subsets; similarly, EV suppressive potential towards NK and T cell proliferation was proportional to their uptake by those cell types. Notably, by using unfractionated PBMCs, we found that EVs released by MSCs were almost entirely incorporated by monocytes, thus lowering the effect on lymphocyte populations. Conversely, purified IECs previously selected for viability and responsiveness to specific stimuli (>90% and >70%, respectively) exhibited a higher degree of EV uptake. These results allowed us to better characterize the EV modulatory effect on each IEC subset and inside the different proliferating IEC generations. The assessment of EV concentration used for the immunological assays was fundamental. To this aim, FACS-based EV quantification resulted in a reliable and accurate method not only to develop standardized immunological methods, but also in view of future clinical application.

Inflammatory cytokines affect dramatically MSC immunosuppression by influencing their secretoma and interactions with IECs both *in vitro* and *in vivo*, thus affecting the clinical effects of MSCs through a functional shift from supportive to inhibitory behavior^7^. Some authors previously reported that IFN-γ-treated MSCs display different protein content inside EVs as compared to resting MSCs, and this phenomenon has an impact on their protective potential against ischemic acute kidney injury^42^. However, the effect of inflammatory priming on EV-mediated immunosuppression has been never reported so far. Here, we have better highlighted the role of IFN-γ and TNF-α on the release and effect of MSC-derived EVs. MSCs displayed a lower rate of EV release following inflammatory priming; nevertheless, primed EVs were more internalized by IECs, probably because of their higher expression of ICAM-1, a molecule involved in the tethering between EVs and target cells^28^. Furthermore, inflammatory priming enhanced EV immunosuppressive effect, mostly in presence of B cells. Thus, at least two different immunological activities of MSC-derived EVs were observed: the first is the EV intrinsic capability to suppress, rather than support, B and NK cells; the other effect is their capability to induce MSC licensing, as primed EVs enhanced MSC regulatory properties. Despite MSC-derived EVs did not inhibit directly T cell proliferation, a significant rescue of T cell proliferation in presence of primed (inhibitory) MSCs was observed when exosome production was prevented by a specific inhibitor (GW4869). In addition, resting MSCs activated with primed EVs showed a significant increase in their immunosuppressive properties, and this effect was observed only in presence of T cells. Overall, we assume that primed EVs could make MSCs to become immunosuppressive towards T cells, a mechanism that would appear to be mediated by IDO increase. Our results are in agreement with the previous evidence that MSC-derived exosomes may polarize monocytes towards M2-like phenotype, which in turn induces CD4^pos^ T cell differentiation towards T regulatory cells^57^, and with other reports showing that IFN-α or -γ transfer via exosomes triggers immunological molecular pathways into target cells^58^,^59^. Moreover, similarly to DC-derived exosomes^33^, miRNA-155 and miRNA-146 were detected inside EVs delivered by MSCs. Interestingly, MSC licensing triggered this mechanism: following cytokine activation, MSCs significantly enhanced the expression and the release of these immunomodulatory molecules within EVs. These data suggest a hypothetical involvement of EV-derived miRNAs both in the direct effect of EVs on IEC proliferation and in their capability to increase MSC priming. Specific experiments are currently ongoing in our lab to better understand the molecular pathways involved in the miRNA mediated immunomodulation.

The *in vitro* immunological features of MSC-derived EVs here described are consistent with their beneficial effects observed *in vivo* in different inflammatory diseases^26^,^34^,^35^,^37^,^60^, including refractory GvHD^61^. Our group contributed recently to show that the clinical improvement observed in two patients with severe refractory acute respiratory distress syndrome (ARDS) following MSC administration was likely mediated by the secretion of EVs containing several proteins known to be involved in the therapeutic effect in other disease models^62^. The *in vivo* administration of MSC-derived EVs could reduce inflammation inside damaged organs and make resident MSCs immune regulatory cells.

## Conclusion

Our study provides new evidence on the immunological activity of MSC-derived EVs towards different IECs and the usefulness of quantitative and reproducible immunological assays to characterize MSC-derived EV immunomodulation. However, the effects observed are less dramatic than those exerted by entire MSCs; consequently, *in vivo* studies are mandatory to evaluate the potential clinical benefit of using EVs rather than *ex-vivo* expanded, clinical grade MSCs.

## Additional Information

**How to cite this article**: Di Trapani, M. *et al.* Differential and transferable modulatory effects of mesenchymal stromal cell-derived extracellular vesicles on T, B and NK cell functions. *Sci. Rep.* **6**, 24120; doi: 10.1038/srep24120 (2016).

## Supplementary Material

Supplementary Information
