# Supplementary material for: Differential and transferable modulatory effects of mesenchymal stromal cell-derived extracellular vesicles on T, B and NK cell functions
Source: Sci Rep. 2016 Apr 13;6:24120. doi: 10.1038/srep24120 (PMC4829861; doi:10.1038/srep24120)
Supplement: Supplementary Information [file srep24120-s1.pdf]

Differential and transferable modulatory effects of mesenchymal stromal cell-derived extracellular vesicles on T, B and NK cell functions

Mariano Di Trapani[1♯], Giulio Bassi[1♯], Martina Midolo[1], Alessandro Gatti[1], Paul Takam[1], Adriana Cassaro[1], Roberta Carusone[1], Annalisa Adamo[1], and Mauro Krampera[1*].

[1] Stem Cell Research Laboratory, Section of Hematology, Department of Medicine, University of Verona, Italy

[♯]MDT and GB contributed equally to this work

***Corresponding author:**

Mauro KRAMPERA, MD, PhD

Stem Cell Research Laboratory

Section of Hematology, Department of Medicine

University of Verona, Italy

Policlinico "G.B. Rossi", P.le L.A.Scuro, 10

37134 Verona (Italy)

Phone: +39 045 8124173

Fax +39 045 8027488

E-mail: mauro.krampera@univr.it

www.stemcellreslab-verona.it

Supplementary Methods

Western Blot

MSCs and MSC-EV were solubilized in RIPA buffer (50 mM Tris HCl pH 7.2, 1% v/v Sodium Deoxycholate, 1% v/v Triton X-100, 3% v/v SDS, 150 mM NaCl, 1 mM EDTA) with SIGMAFAST^{TM} (Sigma Aldrich) and Sodium orthovanadate (Sigma Aldrich). Then 35µg of MSC and MSC-EV proteins were subjected to 10% gradient SDS-PAGE and then blotted onto nitrocellulose membrane filters (GE Healthcare). The correct transfer was confirmed by stining of the membrane with Ponceau Red. The membranes were blocked with 5% non-fat milk in Tris-buffered saline Tween (TBST) (20 mM Tris-HCl pH 7.5, 150 mM NaCl and 0.1% Tween), except for anti-HSP70, which was blocked with 2% BSA in TBST. Then, the blots were incubated at 4°C overnight with the following primary antibodies anti-human markers: rabbit polyclonal anti-Alix (Novusbio), rabbit monoclonal anti-CD9 (Novusbio), rabbit monoclonal anti-HSP70 (Santa Cruz), rabbit monoclonal anti-LAMP1 (Cell Signaling), rabbit polyclonal anti-GRP78 (Abcam), rabbit polyclonal anti-Giantin (Abcam), mouse monoclonal anti-IDO1 (Novusbio). Next, the blots were washed with TBST and incubated for 1 hour at room temperature with the specific peroxidase-conjugated secondary antibodies (Cell Signaling). After washing with TBST, the proteins were detected by enhanced chemiluminescence (ECL) (Euroclone) and analyzed by LAS 4000 Image analyzer (GE Healthcare).

Real-time PCR Analysis

Total RNA was extracted from MSCs and EVs using miRNeasy Mini kit (Qiagen) and converted into cDNA with miScript II RT kit (Qiagen). Real-time qPCR for miRNAs analysis was performed using miScript SYBR Green PCR kit (Qiagen) and specific primers for miRNA-146a-5p, miRNA-146b-5p and miRNA-155-5p (miScript Primer Assays, Qiagen). IDO expression was detected using the Platinum SYBR Green qPCR SuperMix-UDG (Life Technologies). Primer sequences for IDO1 gene were forward 5'-TTGTTCTCATTTCGTGATGG-3' and reverse 5'-TACTTTGATTGCAGAAGCAG-3', while for GAPDH gene were forward 5'-ACAGTTGCCATGTAGACC-3' and reverse 5'-TTTTTGGTTGAGCACAGG-3' (all from Sigma Aldrich). RNU6-2 (miScript Primer Assays, Qiagen) and GAPDH were used as internal controls to normalize the relative expression of miRNAs and IDO, respectively. Real-time qPCR was conducted in triplicate in 96-well plate using Lightcycler 480 (Roche), and the relative RNA expression was calculated according to the $\Delta\Delta CT$ method.

MicroRNA analysis

The Agilent 2100 Bioanalyzer (Agilent) were used to assess the small RNA profiles in MSCs and MSC-EVs. Arrays were performed on three different RNA preparations from resting and primed MSCs and their EVs.

Supplementary Figures

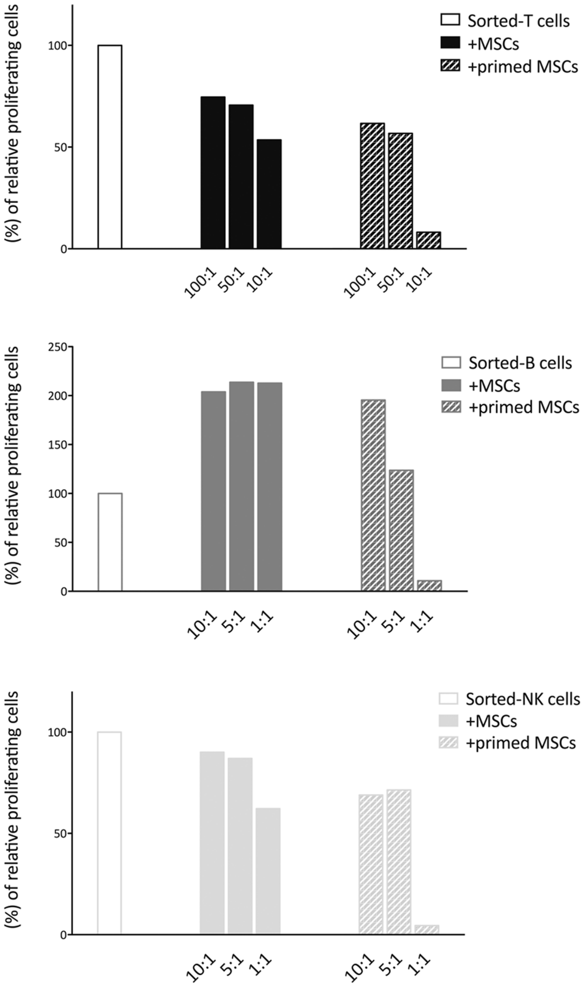

Supplementary Fig. S1. **Ratio-dependent immunomodulatory effect of MSCs on sorted-IECs**. Sorted T, B and NK cells were cultured in presence of indicated amount of MSCs. CFSE fluorescence was analyzed after 6 days for T and NK cells and after 4 days for B cells. The results are expressed as relative proliferation percentage of IECs, normalized to IEC cultured alone (100%).

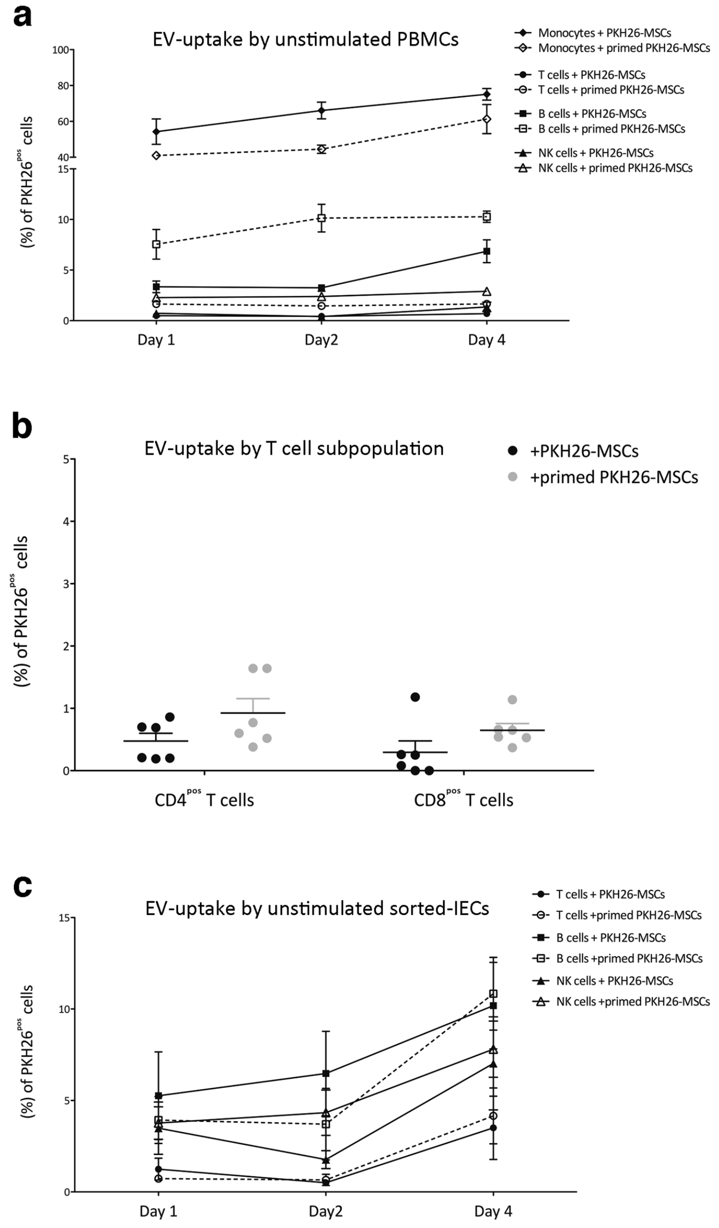

Supplementary Fig. S2. **Time course of EV-uptake by IECs**. Resting and primed PKH26-MSCs were cultured in presence of unfractionated PBMCs (a) or sorted T, B and NK cells (c). After 1, 2 and 4 days, cells were harvested and labeled with lineage specific antibodies (as described in Materials and Methods section). PKH26-EV uptake in $CD4^{pos}$ and $CD8^{pos}$ PBMCs (b). The EV-uptake by IECs was detected as

percentage of lineage specific^{pos}/PKH26^{pos} IECs by FACS. Error bars represented mean ± SEM of three independent experiments.

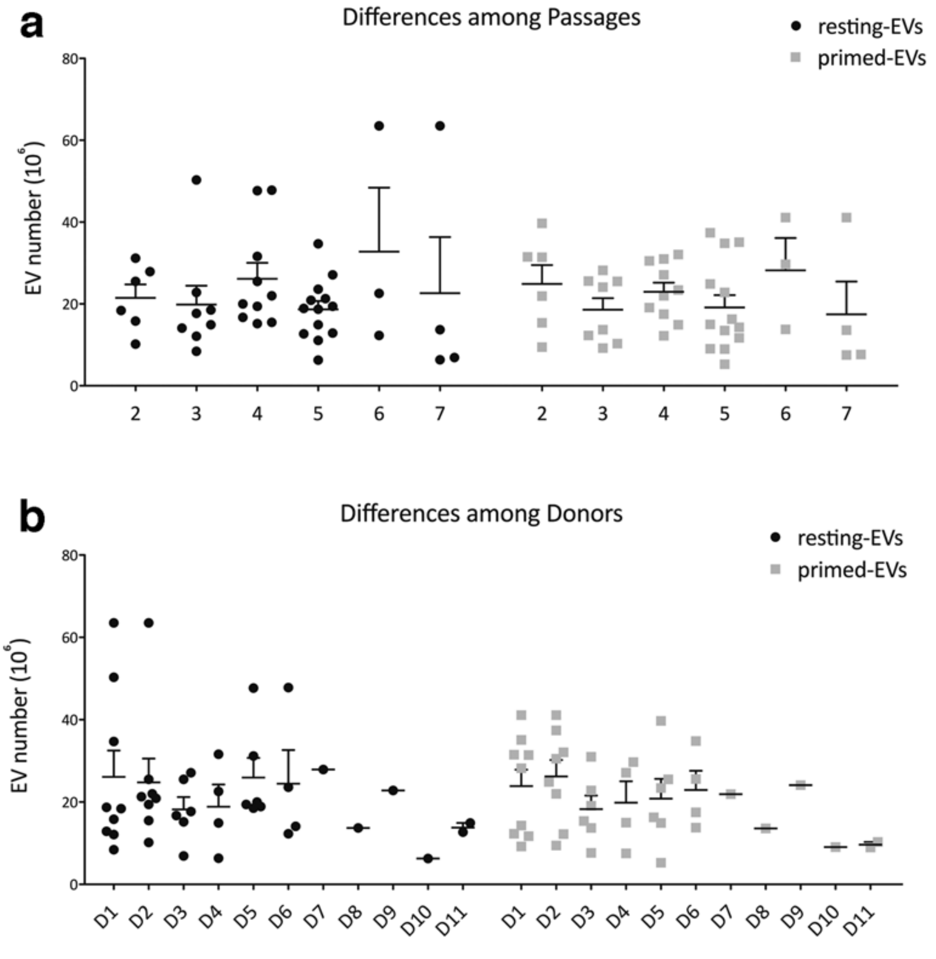

Supplementary Fig. S3. Quantitative differences among EVs purified from MSCs derived from different cell passages (a) and donors (b). EV number was calculated using TruCount beads and the absolute count was subtracted to background noise events from 0.22 µm-filtered PBS, as described in Materials and Methods.

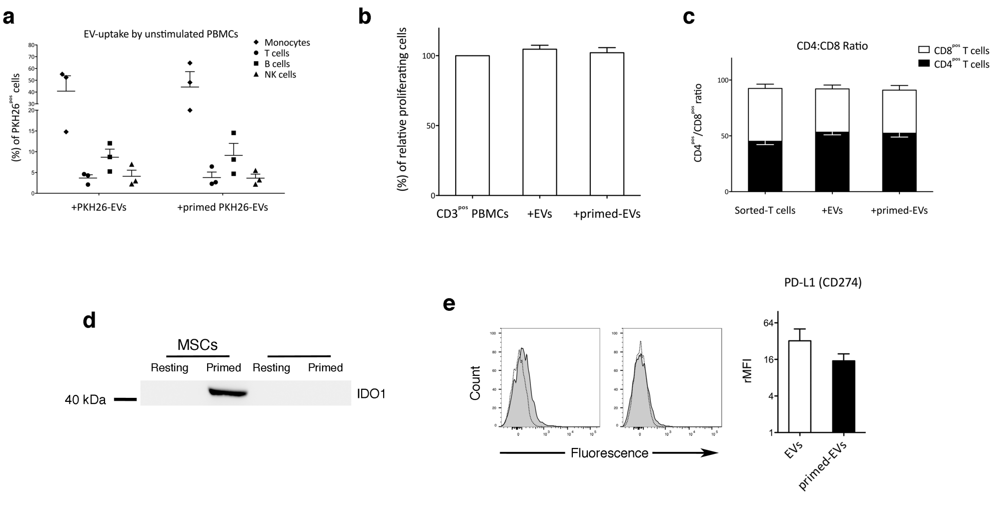

Supplementary Fig. S4. (a) Purified PKH26-EVs were isolated from resting and primed MSCs and cultured in presence of unstimulated PBMCs or sorted-T, -B or -NK cells in order to assess the transfer of MSC-derived EVs to IECs. After 4 days, the cells were harvested and labeled with anti-CD45, anti-CD3, anti-CD14, anti-CD56, anti-CD19 to identify the different IEC lineage inside unfractionated PBMCs. Effect of resting and primed EVs on CD3^{pos} PBMC proliferation (b) and on CD4 or CD8 T cell subpopulation (c). EVs were added to stimulated PBMCs or sorted-T cells ($1x10^4$ /$3x10^6$ PBMC/EV ratio) and after 4 days cells were harvested and analyzed by FACS. (d) Immunoblot analysis of IDO1 expression in resting and primed MSCs and their purified EVs. This blot is representative of three independent experiments showing the same trends. (e) Immunophenotypic analysis of MSC and their EVs showing the expression of PD-L1 express as the difference of geometric mean fluorescence intensity (rMFI) obtained for each marker and its isotype-matched negative control. Error bars represented mean ± SEM of four independent experiments. ***P < 0.001.

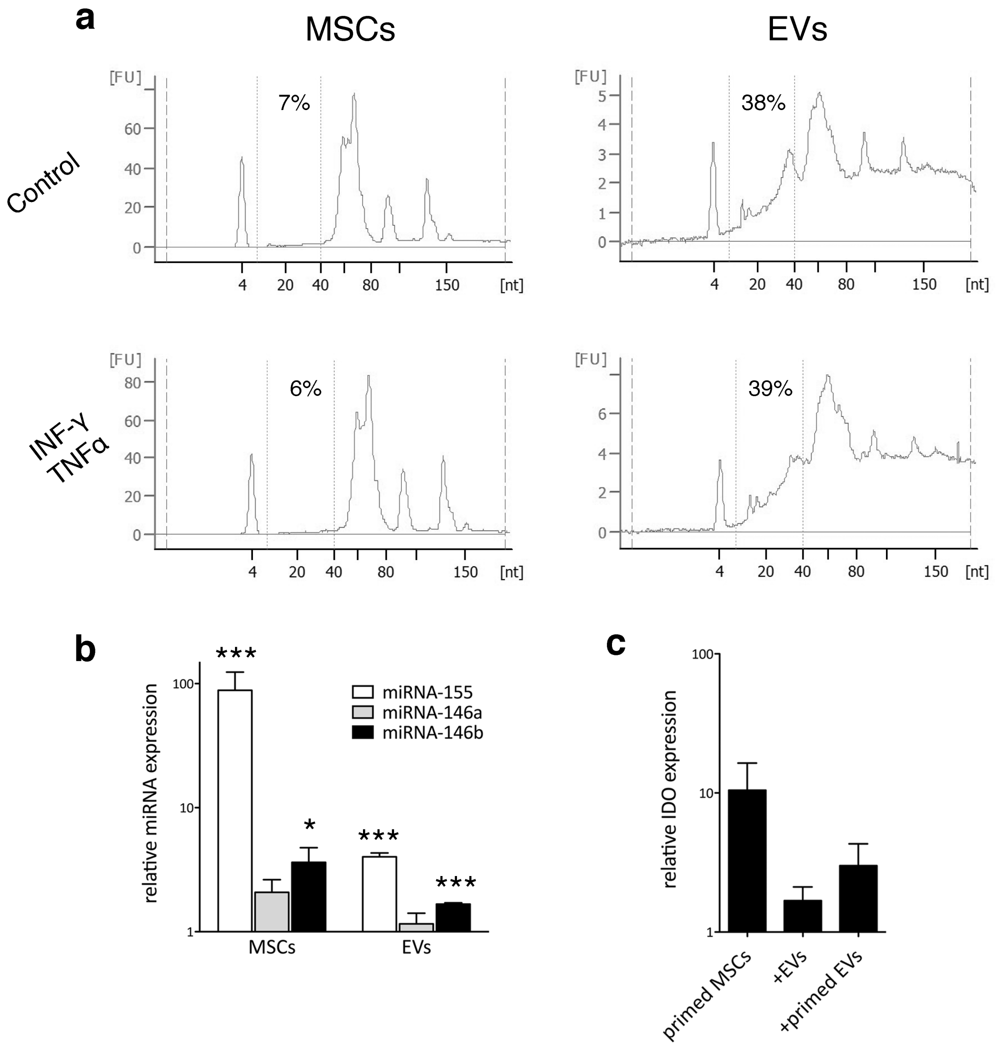

Supplementary Fig. S5. (a) RNA from resting and primed MSCs and their EVs was assessed with the Agilent 2100 Bioanalyzer. The percentage of small RNAs (<40 nucleotides) was higher in EVs than in their donor cells. FU, fluorescence units; nt, nucleotides. (b) Real-Time PCR was used to measure relative miRNA-155, miRNA-146a and miRNA-146b levels in resting and primed MSCs and their EVs. (c) Real-Time analysis was used to assess the modulation of IDO expression in MSCs treated with inflammatory cytokines, resting or primed EVs. Error bars represented mean ± SEM of three independent experiments. *P < 0.05, ***P < 0.001.
